# An Alternate Technique for Goniotomy

**DOI:** 10.18502/jovr.v17i2.10785

**Published:** 2022-04-29

**Authors:** Kouros Nouri-Mahdavi

**Affiliations:** ^1^Director, Glaucoma Advanced Imaging Laboratory, Glaucoma Division, Stein Eye Institute, Department of Ophthalmology at David Geffen School of Medicine, Los Angeles CA

Trabeculotomy as a surgical option has a longstanding history in the field of glaucoma. Performing trabeculotomy in adult eyes has taken a new life since the introduction of TrabectomeⓇ followed by various other approaches for tearing or excising the trabecular meshwork. Shute and colleagues report in this issue of the *Journal of Ophthalmic and Vision Research* the results of a novel low-cost approach for performing trabeculotomy and removal of trabecular meshwork with a 25G hypodermic needle, which they call BANG (bent ab interno needle goniectomy).^[1]^ The premise is that this simple and low-tech approach can remove the trabecular meshwork and reduce the IOP as well as existing techniques such as goniotomy with Kahook Dual Blade (KDB) or trabeculotomy with Trabectome, which are costly due to the need for special instrumentation.

This low-cost approach seems to be effective and promising based on the preliminary six-month results in 41 eyes. The authors do provide histopathological evidence that a strip of TM tissue can be removed with BANG, however, it is not clear how easy and consistent BANG is to remove the TM in every patient using this method. An average of 102º of trabeculotomy was performed in the patient cohort, likely a reflection of the efficacy and simplicity of this approach. The average ( ± SD) maximum intraocular pressure (IOP) was 23.9 ( ± 6.1) mmHg in the study cohort; it is not clear though if this represents untreated maximum IOPs. Also, there are no data on washed-out IOPs after surgery. The average preoperative IOP was 17.4 ( ± 4.1) mmHg on 1.1 ( ± 1.4) topical glaucoma medications, which decreased to 13.3 ( ± 2.5 mmHg) at month six after the intervention; it must be noted that only 16 out of 22 eyes were receiving eye drops for IOP reduction before surgery.

The results seem to be consistent with prior publications on goniotomy with KDB or Trabectome,^[2,3,4]^ however, further experience is needed to confirm the utility of this approach. A similar procedure called trabeculotomy with microhook has been reported by Japanese investigators with seemingly similar success rates as KDB goniotomy combined with phacoemulsification.^[2,3]^ Various techniques are currently used for carrying out trabeculotomy with or without trabecular excision in glaucoma patients. Overall, it appears that trabecular excision may not be necessary to achieve IOP reduction although healing of the trabecular meshwork after trabeculotomy is not well studied and being able to actually remove the roof the Schlemm's canal might contribute to better long-term outcomes.

Before generalizing the conclusions, the characteristics of the study cohort need to be considered. This cohort of patients represents a group of eyes with early to moderate glaucoma with a low medication burden. Despite or maybe due to these features, a 20% or higher IOP reduction was achieved in 73% of patients and 73% of patients had a decrease in eyedrop burden by one or more medication. Six months after surgery, 73% of patients did not need any medication for IOP control. A six-month follow-up period is very short when considered within the lifespan of a glaucoma patient and longer follow-up is certainly required to establish the longevity of the IOP reduction. I will be looking forward to reports on longer term results of this or larger cohorts utilizing BANG to improve patient outcomes especially in conjunction with cataract surgery. The authors are to be commended for attempting to make this type of surgery more accessible to clinicians and patients across the globe.

##  Financial Support and Sponsorship

None.

##  Conflicts of Interest

None declared.
